# Global Routine Vaccination Coverage, 2018

**DOI:** 10.15585/mmwr.mm6842a1

**Published:** 2019-10-25

**Authors:** Megan Peck, Marta Gacic-Dobo, Mamadou S. Diallo, Yoann Nedelec, Samir S. Sodha, Aaron S. Wallace

**Affiliations:** ^1^Epidemic Intelligence Service, CDC; ^2^Global Immunization Division, Center for Global Health, CDC; ^3^Department of Immunization, Vaccines and Biologicals, World Health Organization, Geneva, Switzerland; ^4^Division of Data, Research and Policy, United Nations Children’s Fund, New York City, New York.

Endorsed by the World Health Assembly in 2012, the Global Vaccine Action Plan 2011–2020 (GVAP) (*1*) calls on all countries to reach ≥90% national coverage with all vaccines in the country’s national immunization schedule by 2020. Building on previous analyses (*2*) and using the World Health Organization (WHO) and United Nations Children’s Fund (UNICEF) global vaccination coverage estimates as of 2018, this report presents global, regional, and national vaccination coverage estimates and trends, including vaccination dropout rates. According to these estimates, global coverage with the first dose of diphtheria and tetanus toxoids and pertussis-containing vaccine (DTP1) remained relatively unchanged from 2010 (89%) to 2018 (90%). Global coverage with the third DTP dose (DTP3) followed a similar global trend to that of DTP1, remaining relatively consistent from 2010 (84%) to 2018 (86%) (*3*). Globally, 19.4 million children (14%) were not fully vaccinated in 2018, and among them, 13.5 million (70%) did not receive any DTP doses. Overall, dropout rates from DTP1 to DTP3 decreased globally from 6% in 2010 to 4% in 2018. Global coverage with the first dose of measles-containing vaccine (MCV1) remained between 84% and 86% during 2010–2018. Among countries that offer a second MCV dose (MCV2) during the second year of life, coverage increased from 19% in 2007 to 54% in 2018; among countries offering MCV2 to older age groups (children aged 3–14 years), coverage also increased, from 36% in 2007 to 69% in 2018 (*3*). Globally, the estimated difference in coverage with MCV1 and MCV2 in 2018 was 17%. However, among new and underused vaccines, global coverage increased from 2007 to 2018 for completed series of rotavirus vaccine, pneumococcal conjugate vaccine (PCV), rubella vaccine, *Haemophilus influenzae* type b vaccine (Hib), and hepatitis B vaccine (HepB). To reach global vaccination coverage goals for vaccines recommended during childhood, adolescence, and adulthood, tailored strategies that address local determinants for incomplete vaccination are needed, including targeting hard-to-reach and hard-to-vaccinate populations.

Since the establishment of WHO’s Expanded Programme on Immunization in 1974 to ensure access to Bacille Calmette-Guérin vaccine (BCG), DTP, polio vaccine (Pol), and MCV, an increasing number of vaccines and doses have been introduced (*4*). However, some of these vaccines are recommended after the first birthday; this has added complexity to immunization programs, which typically targeted children during the first year of life. To estimate national vaccination coverage, WHO and UNICEF annually review all available country data, including administrative and survey-based coverage* (*5*,*6*). In general, only doses administered through routine immunization visits (i.e., not those administered through mass vaccination campaigns) are counted. DTP3 coverage by age 12 months is a principal indicator of immunization program performance. Children who have received no doses of DTP are considered to be “left out” of the immunization program; those who received DTP1 but did not complete the series are considered to have “dropped out.” DTP1-to-DTP3 dropout is calculated as the percentage of children who received DTP1 but did not receive DTP3. Because MCV2 is administered during the second year of life, the 2 MCV doses are administered to different birth cohorts; therefore, rather than dropout rates, the percentage point differences in coverage with MCV1 and MCV2 were calculated. To assess missed opportunities for vaccination, differences in vaccination coverage were estimated between selected new and underutilized vaccines (e.g., HepB birth dose, PCV, and rotavirus vaccines) recommended for administration at the same ages as BCG and DTP3.

In 2018, DTP1 coverage ranged from 84% in the African Region to 97% in the European Region. DTP3 coverage followed similar regional trends as those for DTP1, with estimates ranging from 76% in the African Region to 94% in the European Region (Table 1). Overall, 129 (66%) of the 194 WHO member countries achieved ≥90% national DTP3 coverage in 2018, up from 123 (63%) countries in 2017 (*3*). Among the 19.4 million children worldwide who did not complete the 3-dose DTP series in 2018, 13.5 million (70%) received zero DTP doses, and 5.9 million (30%) started but did not complete the DTP series; the overall DTP1-to-DTP3 dropout rate was 4%. Dropout rates varied by region, vaccine, World Bank economic classification,^†^ and eligibility for support from Gavi, the Vaccine Alliance^§^ (Table 2). The 2018 DTP1-to-DTP3 dropout rates ranged from 1% in the Western Pacific Region to 10% in the African Region. DTP1-to-DTP3 dropout rates were highest (7%) among low-income countries and lowest among high-income countries (3%). DTP1-to-DTP3 dropout rates include both populations that are hard to reach and those that are hard to vaccinate. Hard-to-reach populations include those facing supply-side barriers to vaccination because of factors such as geographic distance or terrain, whereas hard-to-vaccinate populations include those who are reachable but whose distrust, religious beliefs, or other factors can lead them to decide against vaccination for their children (*7*).

**TABLE 1:** Coverage with vaccines administered through routine immunization programs,* by vaccine and World Health Organization region — worldwide, 2018

Vaccine	No. (%) of countries with vaccine in schedule	WHO region						
		Total (worldwide)	African	Americas	Eastern Mediterranean	European	South-East Asia	Western Pacific
BCG	156 (80)	**89**	80	91	87	93	91	96
DTP1	194 (100)	**90**	84	92	87	97	92	94
DTP3	194 (100)	**86**	76	87	82	94	89	93
HepB birth dose	108 (56)	**42**	4	68	33	39	48	83
HepB third dose	189 (97)	**84**	76	81	82	84	89	90
Hib3	191 (98)	**72**	76	87	82	76	87	23
MCV1	194 (100)	**86**	74	90	82	95	89	95
MCV2	173 (89)	**69**	26	82	74	91	80	91
PCV3	144 (74)	**47**	73	82	53	78	17	13
Pol3	194 (100)	**85**	74	87	82	93	89	95
RCV1	170 (88)	**69**	32	90	45	95	83	94
Rota_last	101 (52)	**35**	48	73	47	25	24	1

**Abbreviations:** BCG = Bacille Calmette-Guérin vaccine; DTP3 = third dose of diphtheria and tetanus toxoids and pertussis-containing vaccine; HepB = hepatitis B vaccine; Hib3 = third dose of *Haemophilus influenzae* type b vaccine; MCV1 = first dose of measles-containing vaccine; MCV2 = second dose of measles-containing vaccine; PCV3 = third dose of pneumococcal conjugate vaccine; Pol3 = third dose of polio vaccine; RCV1 = first dose of rubella-containing vaccine; Rota_last = final dose of rotavirus vaccine series (number of doses to complete the series varies among vaccine products).

* BCG coverage is based on 156 countries with BCG in the national schedule, whereas coverage for all other vaccines is based on 194 countries worldwide or all countries in the specified region. Administrative coverage is the number of vaccine doses administered to persons in a specified target group divided by the estimated target population. Doses administered during routine immunization visits are counted, but doses administered during supplemental immunization activities (mass campaigns) usually are not. During vaccination coverage surveys, a representative sample of households is visited and caregivers of children in a specified target age group (e.g., aged 12–23 months) are interviewed. Dates of vaccination are transcribed from the child's home-based record, recorded according to caregiver recall, or transcribed from health facility records. Survey-based vaccination coverage is calculated as the proportion of persons in a target age group who received a vaccine dose.

**TABLE 2:** Differences in vaccination coverage for selected vaccine doses given during the first year of life or recommended at the same age, by World Health Organization (WHO) region, Gavi eligibility, and economic classification — worldwide, 2018

Country grouping	Total no. of countries	DTP1 to DTP3 dropout, %*^,†^	DTP3 to PCV3 difference, %^§,¶^	MCV1 to MCV2 difference, %^§,¶^	BCG to HepB birth dose difference, %^§,¶^	DTP3 to Rota_last difference, %^§,¶^	DTP3 to Pol3 difference, %^†,§^
**Total worldwide**	**194**	**4**	**39**	**17**	**47**	**51**	**1**
**WHO region**							
**African**	47	10	3	48	76	28	2
**Americas**	35	5	5	8	23	14	0
**Eastern Mediterranean**	21	6	29	8	54	35	0
**European**	53	3	16	4	54	69	1
**South-East Asia**	11	3	72	9	43	65	0
**Western Pacific**	27	1	80	4	13	92	−2
**Gavi-eligible countries**							
**Worldwide**	68	7	33	26	61	42	0
Economic classification**							
**Low-income country**	30	7	10	46	81	25	2
**Middle-income country**	107	4	49	12	38	57	0
**High-income country**	57	3	5	2	55	46	1

**Abbreviations:** BCG = Bacille Calmette-Guérin vaccine; DTP3 = third dose of diphtheria and tetanus toxoids and pertussis-containing vaccine; HepB = hepatitis B vaccine; MCV1 = first dose of measles-containing vaccine; MCV2 = second dose of measles-containing vaccine; PCV3 = third dose of pneumococcal conjugate vaccine; Pol3 = third dose of polio vaccine; Rota_last = final dose of rotavirus vaccine series (number of doses to complete the series varies among vaccine products).

* Dropout = those who received 1 or 2 DTP doses but did not receive DTP3; calculated using the formula: [(DTP1-DTP3)/DTP1] x 100.

^†^ Only includes countries that have introduced both vaccines and have a WHO/United Nations Children’s Fund (UNICEF) estimate of coverage for both vaccines.

^§^ Difference = percentage point difference between coverage with the first vaccine and the second vaccine (e.g., BCG coverage versus HepB birth dose coverage).

^¶^ Includes countries that have not yet introduced both vaccines or countries that do not have a WHO/UNICEF estimate of coverage for both vaccines.

** Low-income economies are defined as those with a gross national income (GNI) per capita in USD in 2018 of ≤$1,025; middle-income economies are those with a GNI per capita of $1,026–12,375; and high-income economies are those with a GNI per capita of ≥$12,376, calculated using the World Bank Atlas method. https://datahelpdesk.worldbank.org/knowledgebase/articles/906519-world-bank-country-and-lending-groups.

Among the 19.4 million children who failed to receive DTP3 in 2018, 11.7 million (60%) lived in 10 countries, including 5.6 million (29%) who lived in India and Nigeria. Within these 10 countries, among all children who did not receive DTP3, the percentage who failed to receive any DTP doses ranged from 54% to 97%, and the percentage who dropped out between DTP1 and DTP3 ranged from 3% to 46% (Figure).

**FIGURE:**
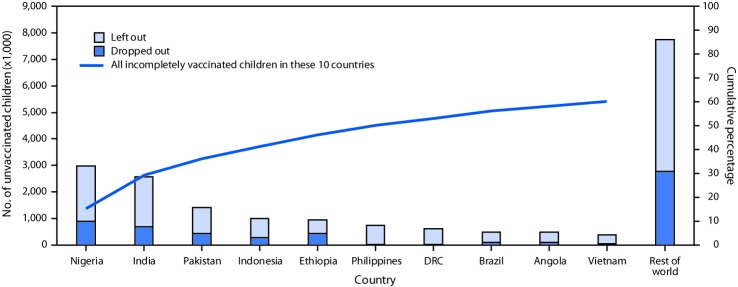
Estimated number of children who were left out* or dropped out^†^ of the immunization program during the first year of life among the 10 countries with the most incompletely vaccinated children and cumulative percentage of all incompletely vaccinated children worldwide accounted for by these 10 countries, 2018 **Abbreviations:** DRC = Democratic Republic of the Congo; DTP1 = 1 dose of diphtheria and tetanus toxoids and pertussis-containing vaccine; DTP3 = third dose of diphtheria and tetanus toxoids and pertussis-containing vaccine. * Never received DTP1. ^†^ Received DTP1 but did not receive DTP3.

In 2018, MCV1 coverage ranged from 74% in the African Region to 95% in the Western Pacific and European regions (Table 1). Globally, 118 (61%) countries achieved the GVAP 2020 target of ≥90% national MCV1 coverage (*1*) in 2018, the same as in 2017. Among all countries, including those that have not yet introduced MCV2, coverage with the second dose by WHO region ranged from 26% in the African Region to 91% in the Western Pacific and European regions (Table 2). Differences in MCV1 and MCV2 coverage varied by region, economic classification, and year of MCV2 introduction. Among regions, the largest difference in coverage between MCV1 and MCV2 was in the African Region (48%), and the smallest (4%) was in the European and Western Pacific regions. By economic classification, the difference in coverage between MCV1 and MCV2 was 46% among low-income countries, 12% in middle-income countries, and 2% in high-income countries. Among the 165 countries that had introduced MCV2 and reported an MCV2 estimate, the largest difference between MCV1 and MCV2 coverage (17%) was estimated among 34 countries that introduced MCV2 during 2010–2017, compared with 5% among 131 countries that introduced the second dose before 2010.

Rotavirus vaccine had been introduced in 101 (52%) countries by 2018. Global coverage with the completed rotavirus series approximately quadrupled, from 8% in 2010 to 35% in 2018. During this period, global coverage also increased for the completed series of PCV (from 11% to 47%), rubella vaccine (35% to 69%), Hib (40% to 72%), and HepB (birth dose: 28% to 42%; 3-dose series: 73% to 84%) (Table 1).

Among all countries (including those that have not introduced the vaccine), the difference in coverage with BCG and HepB birth dose was 47% globally, with the largest difference (76%) in the African Region and the smallest (13%) in the Western Pacific Region (Table 2). The difference between DTP3 and PCV3 coverage was estimated at 39% globally and varied by region, from 3% in the African Region to 80% in the Western Pacific Region. The difference between DTP3 and the final dose of rotavirus vaccine coverage was 51% globally, ranging from 92% in the Western Pacific Region to 14% in the Americas.

## Discussion

Substantial progress has been made in vaccination coverage throughout the world since establishment of the Expanded Programme on Immunization in 1974; in 2018, among countries with available data, 90% of children received at least 1 dose of DTP, and 86% received 3 DTP doses and at least 1 dose of MCV. However, important challenges to achieving high immunization coverage levels for all recommended vaccines remain. Fewer than two thirds of all countries globally reached the GVAP 2020 target of ≥90% national coverage with DTP3 (66%) and MCV1 (61%). Regional differences in vaccination coverage and dropout rates exist, particularly for vaccines offered beyond the first year of life, and need to be addressed through context-specific strategies to reach global, regional, and national immunization coverage goals.

Establishing vaccination contact points during the second year of life and among targeted age groups, including adolescents and pregnant women, is a core component of the GVAP life-course approach. Countries that recently introduced MCV2 into vaccination visits beyond the first year of life still face large gaps in coverage between MCV1 and MCV2. These gaps highlight the challenge of establishing new contact visits and the need for systemic, evidence-informed strategies to address communication and service delivery and improve data systems around vaccine introduction. Recent research highlights the need for a well-organized social mobilization plan targeted to both health care providers and caregivers to ensure that stakeholders understand the importance of these new contact points (*8*). One component of reducing gaps in coverage between vaccines recommended at the same age is elimination of missed opportunities for vaccination; programs should ensure that existing vaccination sites have a secure continuous supply of vaccines and that providers use every health care opportunity to assess vaccination status and administer needed vaccines (*9*). Most African countries (79%) received Gavi funds to support introductions of PCV and rotavirus vaccines; the small differences in the 2018 coverage with these vaccines and DTP3 in the region highlight the importance of this support.

The findings in this report are subject to at least three limitations. First, limitations in data quality (e.g., inaccuracies in vaccination coverage reporting at lower administrative levels and target population information) can result in inaccurate estimations of administrative vaccination coverage. Second, parental recall errors could affect survey-based estimates of coverage (*5*,*10*). Finally, conflict-affected countries are likely to have limited external evaluation of coverage levels, which could limit the accuracy of coverage estimates.

Tailoring strategies to target hard-to-reach and hard-to-vaccinate populations and strengthening immunization systems for administering vaccines recommended beyond infancy are essential to ensure increases in vaccination coverage and disease reduction. Improvements in infrastructure and capacity should be made to improve data quality, particularly enhancement of timeliness and completeness of reporting. Improving initiation and completion of vaccination series that have already been integrated into vaccine schedules, particularly in the African, Americas, Eastern Mediterranean, and Western Pacific regions, is critical to achieving global immunization goals and disease reduction targets (*8*).

**Summary**

**What is already known about this topic?**

Since 1974, global coverage with vaccines to prevent tuberculosis, diphtheria, tetanus, pertussis, poliomyelitis, and measles has increased from <5% to 86%.

**What is added by this report?**

Global coverage with the third dose of diphtheria and tetanus toxoids and pertussis-containing vaccine has not increased above 86% since 2010. Coverage varies across regions and countries, with lower coverage in lower-income countries.

**What are the implications for public health practice?**

Equitable access to immunization to achieve and sustain high coverage can be enhanced through financial and technical support for program strengthening and vaccine introductions in lower-income settings, community engagement to increase vaccination acceptance and demand, collection and use of vaccination data, and commitment to improving immunization services.

## Footnotes

* For a given vaccine, the administrative coverage is the number of vaccine doses administered to persons in a specified target group divided by the estimated target population. Doses administered during routine immunization visits are counted, but doses administered during supplemental immunization activities (mass campaigns) usually are not. During vaccination coverage surveys, a representative sample of households is visited, and caregivers of children in a specified target age group (e.g., aged 12–23 months) are interviewed. Dates of vaccination are transcribed from the child’s home-based record, recorded according to caregiver recall, or transcribed from health facility records. Survey-based vaccination coverage is calculated as the proportion of persons in a target age group who received a vaccine dose.

† Low-income economies are defined as those with a gross national income (GNI) per capita in USD in 2018 of ≤$1,025; middle-income economies are those with a GNI per capita of $1,026–12,375; and high-income economies are those with a GNI per capita of ≥$12,376, calculated using the World Bank Atlas method. https://datahelpdesk.worldbank.org/knowledgebase/articles/906519-world-bank-country-and-lending-groups.

§ Gavi eligibility includes low- and middle-income countries eligible to receive financial assistance through grants contingent on a country’s GNI per capita. Eligibility is defined as a country’s average 3-year GNI per capita in USD of ≤$1,580. As GNI increases, a country moves through Gavi’s different eligibility phases until reaching the transition phase when GNI exceeds the eligibility threshold. https://www.gavi.org.

## References

[1] World Health Organization. Global vaccine action plan 2011–2020. Geneva, Switzerland: World Health Organization; 2013. https://www.who.int/immunization/global_vaccine_action_plan/GVAP_doc_2011_2020/en/

[2] VanderEnde K, Gacic-Dobo M, Diallo MS, Conklin LM, Wallace AS. Global routine vaccination coverage—2017. MMWR Morb Mortal Wkly Rep 2018;67:1261–4. 10.15585/mmwr.mm6745a2 30439873 PMC6290803

[3] World Health Organization. Immunization, vaccines and biologicals—data, statistics and graphs. Geneva, Switzerland: World Health Organization; 2018. https://www.who.int/immunization/monitoring_surveillance/data/en/

[4] Uwizihiwe JP, Bock H. 40th anniversary of introduction of expanded immunization program (EPI): a literature review of introduction of new vaccines for routine childhood immunization in Sub-Saharan Africa. Edmond, OK: International Journal of Vaccines & Vaccination; 2015. https://medcraveonline.com/IJVV/40th-anniversary-of-introduction-of-expanded-immunization-program-epi-a-literature-review-of-introduction-of-new-vaccines-for-routine-childhood-immunization-in-sub-saharan-africa.html

[5] Burton A, Monasch R, Lautenbach B, WHO and UNICEF estimates of national infant immunization coverage: methods and processes. Bull World Health Organ 2009;87:535–41. 10.2471/BLT.08.053819 19649368 PMC2704038

[6] The World Bank. World Bank country and lending groups. New York, NY: The World Bank; 2016. https://datahelpdesk.worldbank.org/knowledgebase/articles/906519-world-bank-country-and-lending-groups

[7] Ozawa S, Yemeke TT, Evans DR, Pallas SE, Wallace AS, Lee BY. Defining hard-to-reach populations for vaccination. Vaccine 2019;37:5525–34. 10.1016/j.vaccine.2019.06.081 31400910 PMC10414189

[8] World Health Organization. Establishing and strengthening immunization in the second year of life: practices for vaccination beyond infancy. Geneva, Switzerland: World Health Organization; 2018. https://apps.who.int/iris/bitstream/handle/10665/260556/9789241513678-eng.pdf?ua=1

[9] World Health Organization. Missed opportunities for vaccination (MOV) strategy. Geneva, Switzerland: World Health Organization; 2017. https://www.who.int/immunization/programmes_systems/policies_strategies/MOV/en/

[10] World Health Organization. Immunization, vaccines and biologicals—data, statistics and graphs Geneva, Switzerland: World Health Organization; 2016. https://www.who.int/immunization/monitoring_surveillance/en

