# *Erratum:* Vol. 68, No. 42

**DOI:** 10.15585/mmwr.mm6844a4

**Published:** 2019-11-08

In the report “Global Routine Vaccination Coverage, 2018,” on page 937, in the list of authors, the fifth author should have been listed as Samir **V.** Sodha, MD.

